# Characterization of acetic acid-detoxifying *Escherichia coli* evolved under phosphate starvation conditions

**DOI:** 10.1186/s12934-016-0441-7

**Published:** 2016-02-19

**Authors:** Patrice L. Moreau, Laurent Loiseau

**Affiliations:** Laboratoire de Chimie Bactérienne, UMR 7283, Aix-Marseille Université, Marseille, France; Institut de Microbiologie de la Méditerranée, Centre National de la Recherche Scientifique, Marseille, France

**Keywords:** Adaptive evolution, *Escherichia coli*, K^+^ homeostasis, Oxidative stress, Acetic acid stress, Metabolism rerouting, Growth in stationary phase

## Abstract

**Background:**

During prolonged incubation of *Escherichia coli* K-12 in batch culture under aerobic, phosphate (Pi) starvation conditions, excess glucose is converted into acetic acid, which may trigger cell death. Following serial cultures, we isolated five evolved strains in two populations that survived prolonged incubation.

**Methods:**

We sequenced the genomes of the ancestral and evolved strains, and determined the effects of the genetic changes, tested alone and in combination, on characteristic phenotypes in pure and in mixed cultures.

**Results:**

Evolved strains used two main strategies: (1) the constitutive expression of the Trk- and Kdp-dependent K^+^ transport systems, and (2) the inactivation of the ArcA global regulator. Both processes helped to maintain a residual activity of the tricarboxylic acid cycle, which decreased the production of acetic acid and eventually allowed its re-consumption. Evolved strains acquired a few additional genetic changes besides the *trkH*, *kdpD* and *arcA* mutations, which might increase the scavenging of organophosphates (*phnE*^+^, *lapB*, and *rseP*) and the resistance to oxidative (*rsxC*) and acetic acid stresses (e14^−^/*icd*^+^).

**Conclusions:**

Evolved strains rapidly acquired mutations (*phnE*^+^*lapB rpoS trkH* and *phnE*^+^*rseP kdpD*) that were globally beneficial to growth on glucose and organophosphates, but detrimental to long-term viability. The spread of these mutant strains might give the ancestral strain time to accumulate up to five genetic changes (*phnE*^+^*arcA rsxC crfC* e14^−^/*icd*^+^), which allowed growth on glucose and organophosphates, and provided a long-term survival. The latter strain, which expressed several mechanisms of protection against endogenous and exogenous stresses, might provide a platform for producing toxic recombinant proteins and chemicals during prolonged incubation under aerobic, Pi starvation conditions.

## Background

In nature, bacteria are frequently starved for essential nutrients such as phosphate (HPO_4_^2−^, Pi) [1, 2]. When the levels of Pi in the medium decrease below 4 μM, *Escherichia coli* induces the PhoBR regulon, which helps to scavenge low levels of Pi and secondary sources of Pi such as organophosphates and phosphonates [3]. When the cells cannot find a sufficient source of Pi to maintain growth, they enter the so-called stationary phase [4]. The *E. coli* K-12 strain MG1655 incubated in batch culture under aerobic, Pi-limiting conditions (0.1 mM K_2_HPO_4_ and 40 mM glucose) enters stationary phase after 10 h of incubation when Pi levels drop below 1 μM [5].

**Fig. 1:**
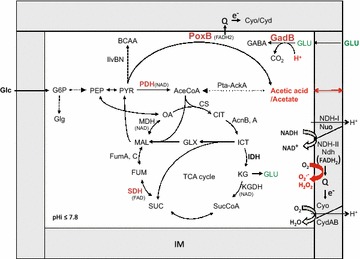
Schematic illustration of the relationship between pyruvate metabolism and the aerobic respiratory chain. *AceCoA* acetyl CoA, *BCAA* branched-chain amino acids, *CIT* citrate, *CS* citrate synthase (GltA), *FAD* flavin adenine dinucleotide, *FUM* fumarate, *GABA* γ-aminobutyrate, *Glc* glucose, *Glg* glycogen, *GLX* glyoxylate, *GLU* glutamate, *G6P* glucose 6-phosphate, *ICT* isocitrate, *IDH* isocitrate dehydrogenase (Icd), *IM* inner membrane, *KG* α-ketoglutarate, *KGDH* α-ketoglutarate dehydrogenase, *MAL* malate, *MDH* malate dehydrogenase, *NDH* NADH dehydrogenase, *OA* oxaloacetate, *PDH* pyruvate dehydrogenase, *pHi* internal pH, *PEP* phosphoenolpyruvate, *PYR* pyruvate, *Q* ubiquinone, *SDH* succinate dehydrogenase, *SUC* succinate, *SucCoA* succinyl CoA; TCA, tricarboxylic acid

At the approach of the stationary phase, the cells steadily accumulate the RpoS (σ^s^) sigma factor [6, 7]. The σ^s^-RNA polymerase holoenzyme poorly transcribes “growth genes” (e.g. genes encoding the succinate dehydrogenase of the tricarboxylic acid cycle) and preferentially transcribes “defense genes” required for the protection of non-growing cells against endogenous stresses such as oxidative stress (e.g. *pdhR*, *poxB*, and *sodC*) and acetic acid stress (e.g. *gadB*) [4, 8–10]. For instance, cells incubated under Pi starvation conditions continue to metabolize glucose at a reduced rate, but eventually redirect the metabolic flux from the pyruvate dehydrogenase towards the RpoS-dependent pyruvate oxidase, PoxB, which directly converts pyruvate into acetic acid. PoxB (pyruvate:Q reductase), in contrast to the pyruvate dehydrogenase, does not use NAD^+^ as a cofactor, which prevents the adventitious production of O_2_^**.−**^ and H_2_O_2_ by NADH dehydrogenases in the aerobic respiratory chain [4]. Whereas the activity of PoxB protects Pi-starved cells against oxidative stress at the entry into stationary phase, this activity can eventually cause the accumulation of high levels of acetic acid, which decrease the internal pH (pHi), stop metabolism and trigger cell death [4, 10]. Death of Pi-starved cells can be alleviated by the addition of glutamate into the medium, which allows the RpoS-dependent GadB acid resistance system to neutralize acetic acid [5, 10] (Fig. 1).

Whereas *E. coli* is normally doomed to die when glutamate is not added in Pi-limiting medium, evolved strains can sweep populations after generally three serial batch cultures. Variants that survive prolonged incubation exhibit different mutant phenotypes refereed to as RpoS^−^ (colonies grown on LB medium produce low levels of glycogen and exhibit a reduced catalase activity) and Glg^−^ (colonies produce reduced levels of glycogen) [1]. From one evolved population, we isolated three strains exhibiting RpoS^−^ (ENZ1901), Glg^−^ (ENZ1902), and Glg^+^ (ENZ1903) phenotypes. In a parallel-evolved population, which contained a majority of Glg^−^ mutants and eventually Glg^+^ cells, we isolated two Glg^+^ evolved strains (ENZ1904 and ENZ1905) [1].

Compared to the ancestral strain, the evolved strain ENZ1901 (RpoS^−^) harbors a single-nucleotide deletion (*rpoS*-ΔG214) that inactivates RpoS, and an 8-bp deletion in *phnE* that activates the PhnE permease [1]. The lack of RpoS activity allows a substantial activity of the tricarboxylic acid cycle in Pi-starved cells, which decreases the production of acetic acid and allows its re-consumption (Ace^+^ phenotype) [1, 5, 11, 12]. The PhnE permease can scavenge organophosphates excreted into the medium [1]. The combination of the *phnE* and *rpoS* evolved alleles triggers a novel growth-under-Pi-starvation (GPS) phenotype, which refers to the ability of a mutant strain—incubated for 1 day in Pi-limiting medium and added in minority into a 1-day-old culture of the ancestral strain in Pi-limiting medium—to resume growth between days 1 and 4 of incubation by using primarily organophosphates released into the mixed culture by the ancestral strain [1]. The GPS phenotype may account at least in part for the spread of the evolved strain ENZ1901 in populations starved for Pi.

If the presence of an *rpoS*-null mutation may help to explain the behavior of the evolved strain ENZ1901 (RpoS^−^), the majority of the evolved strains did not exhibit an RpoS-negative phenotype [1]. This prompted us to determine the complete genotypes, the metabolic patterns and the growth capacities in Pi-limiting medium of the evolved and reconstructed mutant strains. We show here that mutants could overtake populations and detoxify acetic acid by using two novel strategies: (1) K^+^ accumulation (triggered by *trkH* or *kdpD* mutations) combined with changes in outer membrane permeability (*lapB* or *rseP* mutations), and (2) inactivation of ArcA activity (*arcA* mutation) combined with an increased resistance to oxidative stress (*rsxC* mutation) and to acetic acid stress (*crfC* and e14^**−**^**/***icd*^**+**^ mutations).

## Results

### Genotype of the evolved strains

**Fig. 2:**
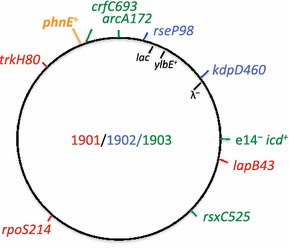
Genetic map of the evolved strains ENZ1901, ENZ1902, and ENZ1903

Whole-genome sequencing of the evolved strains ENZ1901-ENZ1905 and of the ancestral strain ENZ535 revealed limited changes compared to the MG1655 reference sequence (F^−^ λ^−^*rfb*-50 *rph*-1 *ilvG phnE ylbE icd*::e14) (ftp://ncbi.nlm.nih.gov/blast/executables/blast+/LATEST/) (Table 1; Fig. 2):

**Table 1:** *E. coli* K-12 strains

ENZ	Genotype	Source (reference)
535	F^−^ λ^−^ *rfb*-*50 rph*-*1 ilvG phnE ylbE* ^+^ *icd*::e14	[10]
1734	As 535 but Δ*lacIZ*	[10]
1766	As 1734 (535 Δ*lacIZ*) but *poxB176*::*lacZ.Cm* ^*r*^	[10]
1791	As 535 but Δ*lacY*::*kan*	[1]
1797	As 535 but *lacY*::Tn*10*	[1]
1832	Δ*lacX74* (λ *rpoH*P3::*lacZ*)	SEA001 [7]
1833	Δ*lacU169* (λ *cydA*::*lacZ*-*km*)	ASA12 [13]
1842	Δ*lacIZ* (P*kdp*::*lacZ*)	LF3 [14]
1843	Δ*lacU* (λ P*soxS*::*lacZ*)	SP11 [15]
1901	As 535 but *phnE* ^+^ *trkH80 rpoS214 lapB43*	Evolved 535 RpoS^−^ [1]
1902	As 535 but *phnE* ^+^ *kdpD460 rseP98*	Evolved 535 Glg^−^ [1]
1903	As 535 but *phnE* ^+^ *arcA172 rsxC525 crfC693* e14^−^/*icd* ^+^	Evolved 535 Glg^+^ [1]
1904	As 535 but *phnE* ^+^	Evolved 535 Glg^+^ [1]
1905	As 535 but *phnE* ^+^	Evolved 535 Glg^+^ [1]
1944	As 535 but Δ*rpoS*::*kan*	[1]
1945	As 1734 (535 Δ*lacIZ*) but Δ*rpoS*::*kan*	[1]
1946	As 1944 (535 Δ*rpoS*) but Kan^s^	[1]
1947	As 1945 (535 Δ*lacIZ* Δ*rpoS*) but Kan^s^	This study
1982	As 1903 but Δ*lacY*::*kan*	[1]
1984	As 1905 but Δ*lacY*::*kan*	[1]
2000	As 1901 but Δ*lacY*::*kan*	[1]
2001	As 1902 but Δ*lacY*::*kan*	[1]
2003	As 1904 but Δ*lacY*::*kan*	[1]
2005	As 1944 (535 Δ*rpoS*::*kan*) but *cysC95*::Tn*10*	[1]
2020	As 1946 (535 Δ*rpoS*) but Δ*lacY*::*kan*	[1]
2032	As 1901 but Δ*lacIZ*	This study
2033	As 1902 but Δ*lacIZ*	This study
2034	As 1903 but Δ*lacIZ*	This study
2041	As 535 but *rpoS214*	[1]
2043	As 1901 but *rpoS* ^+^ (*phnE* ^+^ *trkH80 lapB43)*	This study
2044	As 1902 (*phnE* ^+^ *kdpD460 rseP98*)	This study
2045	As 1734 (535 Δ*lacIZ*) but *poxB176*::*lacZ.Cm* ^*r*^	P1.1766
2046	As 1947 (535 Δ*lacIZ* Δ*rpoS*) but *poxB176*::*lacZ.Cm* ^*r*^	P1.1766
2065	As 2041 (535 *rpoS214*) but Δ*lacY*::*kan*	[1]
2067	As 2043 (*phnE* ^+^ *trkH80 lapB43*) but Δ*lacY*::*kan*	[1]
2068	As 2044 (*phnE* ^+^ *kdpD460 rseP98*) but Δ*lacY*::*kan*	[1]
2112	As 2044 but Δ*ybfH*::*kan kdpD* ^+^ (*phnE* ^+^ *rseP98*)	[16]
2136	As 2043 (1901 *rpoS* ^+^) but Δ*yihL*::*kan* (*phnE* ^+^ *lapB43 trkH80*)	[16]
2137	As 2043 (1901 *rpoS* ^+^) but Δ*yihL*::*kan trkH* ^+^ (*phnE* ^+^ *lapB43*)	[16]
2145	As 1902 but *lacY*::Tn*10*	This study
2154	As 2044 (1902) but Δ*ybfH*::*kan*	[16]
2163	As 535 but Δ*yihL*::*kan trkH80*	P1.2136
2166	As 535 but Δ*yihL*::*kan trkH* ^+^	P1.2136
2168	As 535 but Δ*ybfH*::*kan kdpD460*	P1.2154
2174	As 535 but Δ*ybfH*::*kan kdpD* ^+^	P1.2154
2263	As 535 but Δ*proP*::*kan phnE* ^+^ *rpoS214*	[1]
2282	As 1901 but *lacY*::Tn*10*	[1]
2309	As 535 but Δ*trkH*::*kan*	[16]
2315	As 535 but *phnE* ^+^	This study
2317	As 2309 (535 Δ*trkH*) but *phnE* ^+^	This study
2326	As 1902 but Δ*yaeH*::*kan*	[16]
2329	As 1734 (535 Δ*lacIZ*) but *phnE* ^+^	This study
2333	As 1832 (*rpoH*P3::*lacZ*) but *phnE* ^+^	This study
2337	As 1842 (P*kdp*::*lacZ*) but *phnE* ^+^	This study
2338	As 2045 (*poxB176*::*lacZ.Cm* ^*r*^) but *phnE* ^+^	This study
2341	As 2329 (535 Δ*lacIZ* *phnE* ^+^) but *poxB176*::*lacZ.Cm* ^*r*^	P1.1766
2343	As 2032 (1901 Δ*lacIZ*) but *poxB176*::*lacZ.Cm* ^*r*^	P1.1766
2345	As 2033 (1902 Δ*lacIZ*) but *poxB176*::*lacZ.Cm* ^*r*^	P1.1766
2347	As 2034 (1903 Δ*lacIZ*) but *cydA*::*lacZ*-*kan*	P1.1833
2354	As 2315 (535 *phnE* ^+^) but Δ*lacY*::*kan*	This study
2355	As 2315 (535 *phnE* ^+^) but *lacY*::Tn*10*	This study
2360	As 2337 (P*kdp*::*lacZ*) but Δ*ybfH*::*kan kdpD460*	P1.2154
2363	As 2337 (P*kdp*::*lacZ*) but Δ*ybfH*::*kan kdpD* ^+^	P1.2154
2395	As 2163 (535 Δ*yihL*::*kan trkH80*) but *phnE* ^+^	This study
2401	As 2315 (535 *phnE* ^+^) but Δ*ybfH*::*kan kdpD* ^+^	P1.2154
2402	As 2315 (535 *phnE* ^+^) but Δ*ybfH*::*kan kdpD460*	P1.2154
2405	As 2315 (535 *phnE* ^+^) but Δ*yihL*::*kan trkH80*	P1.2136
2413	As 535 but Δ*arcA*::*kan*	[16]
2414	As 535 but Δ*rsxC*::*kan*	[16]
2415	As 1903 but Δ*arcA*::*kan*	[16]
2416	As 1903 but Δ*rsxC*::*kan*	[16]
2418	As 2034 (1903 Δ*lacIZ*) but *cydA*::*lacZ*-*kan*	P1.1833
2420	As 1843 (P*soxS*::*lacZ*) but Δ*arcA*::*kan*	[16]
2421	As 1843 (P*soxS*::*lacZ*) but Δ*rsxC*::*kan*	[16]
2424	As 2413 (535 Δ*arcA*) but Kan^s^	This study
2430	As 2420 (P*soxS*::*lacZ* Δ*arcA*) but Kan^s^	This study
2434	As 2424 (535 Δ*arcA*) but Δ*rsxC*::*kan*	[16]
2436	As 2430 (P*soxS*::*lacZ* Δ*arcA*) but Δ*rsxC*::*kan*	[16]
2439	As 2434 (535 Δ*arcA* Δ*rsxC*::*kan*) but *phnE* ^+^	This study
2455	As 2413 (535 Δ*arcA*::*kan*) but *phnE* ^+^	This study
2456	As 2413 (535 Δ*rsxC*::*kan*) but *phnE* ^+^	This study
2480	As 2333 (*rpoH*P3::*lacZ* *phnE* ^+^) but Δ*rseA*::*kan*	[16]
2489	As 2333 (*rpoH*P3::*lacZ* *phnE* ^+^) but Δ*yaeH*::*kan rseP* ^+^	P1.2326
2490	As 2333 (*rpoH*P3::*lacZ* *phnE* ^+^) but Δ*yaeH*::*kan rseP98*	P1.2326
2493	As 2341 (*poxB176*::*lacZ.Cm* ^*r*^) but Δ*yihL*::*kan trkH* ^+^	P1.2136
2494	As 2341 (*poxB176*::*lacZ.Cm* ^*r*^) but Δ*yihL*::*kan trkH80*	P1.2136
2495	As 2341 (*poxB176*::*lacZ.Cm* ^*r*^) but Δ*ybfH*::*kan kdpD* ^+^	P1.2154
2496	As 2341 (*poxB176*::*lacZ.Cm* ^*r*^) but Δ*ybfH*::*kan kdpD460*	P1.2154

**Fig. 3:**
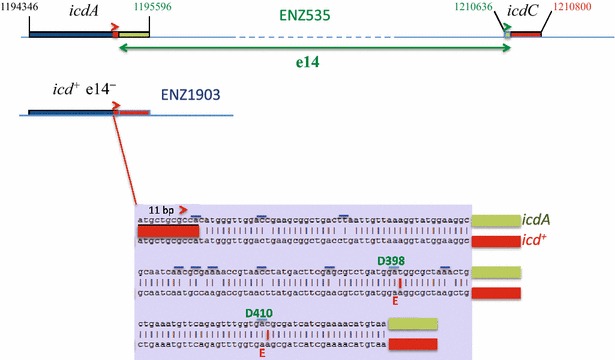
Genetic structures of *icd* alleles. The *icd* sequence in ENZ1903 (e14^−^ */icd* ^+^) contained 12 single-nucleotide polymorphisms in comparison with the reference sequence, *icdA*, in ENZ535 (*icd*::e14). This implicates that a site-specific recombination occurred in the 11-bp direct repeats present in *icdA* and *icdC* (≫), which led to the deletion/excision of the prophage e14. *Blue bars* indicate codons harboring substitutions

1. In the ancestral strain ENZ535, the sequence of the *ylbE* gene differed from the reference sequence by a single-nucleotide polymorphism and a single-nucleotide insertion that removed a stop codon. The so-called *ylbE*^+^ allele in ENZ535 is also present in the MG1655 strain referred to as ATCC47076 [17]. The function of YlbE is not known.
2. In the five evolved strains (PhnE-expressing), the sequence of the *phnE* gene differed from the reference sequence by a deletion of 8 bp, which removed a frameshift inactivating *phnE* [1].
3. The evolved strains ENZ1904 and ENZ1905 (Glg^+^) acquired no others mutations besides the 8-bp deletion in *phnE* (*phnE*^**+**^).
4. The evolved strains ENZ1901 (RpoS^−^), ENZ1902 (Glg^−^), and ENZ1903 (Glg^+^) harbored unique genetic changes scattered around the chromosome (Fig. 2): *rpoS*-ΔG214, *trkH*-L80Q and *lapB*-V43G in ENZ1901; *kdpD*-D460V and *rseP*-A98V in ENZ1902; and *arcA*-F172Y, *rsxC*-I525F, *crfC*-E693Stop and e14^−^*/icd*^+^ in ENZ1903. In the latter strain, excision of the lambdoid prophage e14 restored the *icd*^+^ sequence that differed from the reference sequence, *icdA*, by 12 single-nucleotide polymorphisms [18, 19] (Fig. 3).

Considering the phenotypic and genetic diversity that occurred in evolved populations, a number of questions arise regarding (1) the minimum number of mutations required by a variant to sweep a population [20–23], (2) the types of mutations (loss- or gain-of-function; global regulatory or specific changes) [20–27], and (3) the diversity or convergence of the metabolic changes occurring in evolved strains [28, 29].

### In ENZ1901 (*phnE*^+^*rpoS214 trkH80lapB43*), the *trkH80* and *rpoS214* mutations similarly affect the metabolic flux

To analyze the effects of the *trkH80* and *lapB43* mutations present in the evolved strain ENZ1901 (*phnE*^+^*rpoS214 trkH80lapB43*), we exchanged the *rpoS214* allele for the *rpoS*^+^ allele from the ancestral strain ENZ535, which gave rise to the strain ENZ2043 (*phnE*^+^*trkH80 lapB43*). This strain exhibited a Glg^**−**^ phenotype, whereas strains that harbored a single *phnE*^+^ allele (e.g. ENZ1904) exhibited a Glg^**+**^ phenotype. Thus, besides the *rpoS214* mutation, which decreased the cellular levels of glycogen and of catalase, the *trkH80* and/or *lapB43* mutation(s) somehow decreased the cellular levels of glycogen.

LapB is an inner-membrane protein that plays a key role in the biogenesis of the glycolipid lipopolysaccharide leaflet in the outer membrane [30, 31]; at first glance, it seemed unlikely that a *lapB* mutation might affect the glycogen content, a storage compound. In contrast, changes in the activity of TrkH, the primary potassium (K^+^) transporter, might possibly change the levels of glycogen because the deregulation of K^+^ homeostasis, which normally occurs in response to an osmotic stress, affects many metabolic processes [32].

To determine whether mutations in *trkH* could change the Glg phenotype, we transduced the Δ*trkH*::*kan* deletion and the *trkH80* evolved mutation (co-transduced with Δ*yihL*::*kan*) into the ancestral strain ENZ535. Compared to the parental strain, the Δ*trkH* (ENZ2317) and *trkH80* (ENZ2163) mutant strains produced more and less glycogen, respectively. Considering that these opposite effects on glycogen contents might reflect changes in K^+^ transport, and that the rate of transport of K^+^ is decreased in Δ*trkH* mutants [32], we concluded that the rate of transport of K^+^ could be increased in *trkH80* mutants. The TrkH-L80Q mutant protein might therefore mimic the form of TrkH (or of the TrkAEGH complex) that is normally present following an osmotic stress, when the rate of transport of K^+^ through TrkH transiently increases [32].

Changes in K^+^ levels may affect the selectivity of sigma factors [32, 33]. To account for the Glg^**−**^ phenotype of a *trkH80* mutant strain, a simple hypothesis was that excess K^+^ might partially decrease the activity of RpoS, which would reduce the synthesis of glycogen [25]. To test this hypothesis, we used the *poxB176*::*lacZ*.Cm^r^ fusion as a reporter of the RpoS activity, and measured the β-galactosidase activity in 1-day old cultures in P- and N-limiting media [10]. The expression of *poxB* is strictly controlled by the activity of RpoS, which increases poorly in N-starved cells and strongly in P-starved cells [4, 34]. As shown in Table 2, the *poxB176*::*lacZ* fusion was barely expressed in strains carrying *rpoS*-null mutations: ENZ2046 (Δ*rpoS*) and ENZ2343 (*rpoS214 trkH80 lapB43*). In contrast, the *poxB176*::*lacZ* fusion was normally induced (moderately in N- and strongly in P-limiting media) in the strains ENZ2493 and ENZ2494 that harbored the *trkH*^+^ and *trkH80* alleles, respectively. Therefore, the *trkH80* mutation had by itself no effect on the activity of RpoS in Pi-starved cells.

**Table 2:** RpoS activity measured with the levels of expression of the *poxB176*::*lacZ* fusion in strains starved for N or P

ENZ strains	β-Galactosidase (units)	
	24 h in N-limiting medium	24 h in P-limiting medium
2045: *rpoS* ^+^	104 ± 1 (n = 3)	439 ± 25 (n = 3)
2046: Δ*rpoS*	1 ± 0.2 (n = 2)	4 ± 1 (n = 2)
2343: *rpoS214 trkH80 lapB43*	1 ± 0.2 (n = 2)	3 ± 0.4 (n = 2)
2493: *yihL*::*kan* *trkH* ^+^	108 ± 5 (n = 3)	437 ± 2 (n = 3)
2494: *yihL*::*kan* *trkH80*	104 ± 4 (n = 2)	404 ± 64 (n = 2)
2345: *kdpD460* *rseP98*	109 ± 10 (n = 2)	435 ± 5 (n = 2)
2495: *ybfH*::*kan* *kdpD* ^+^	112 ± 2 (n = 3)	430 ± 63 (n = 3)
2496: *ybfH*::*kan* *kdpD460*	113 ± 6 (n = 3)	469 ± 88 (n = 3)

**Fig. 4:**
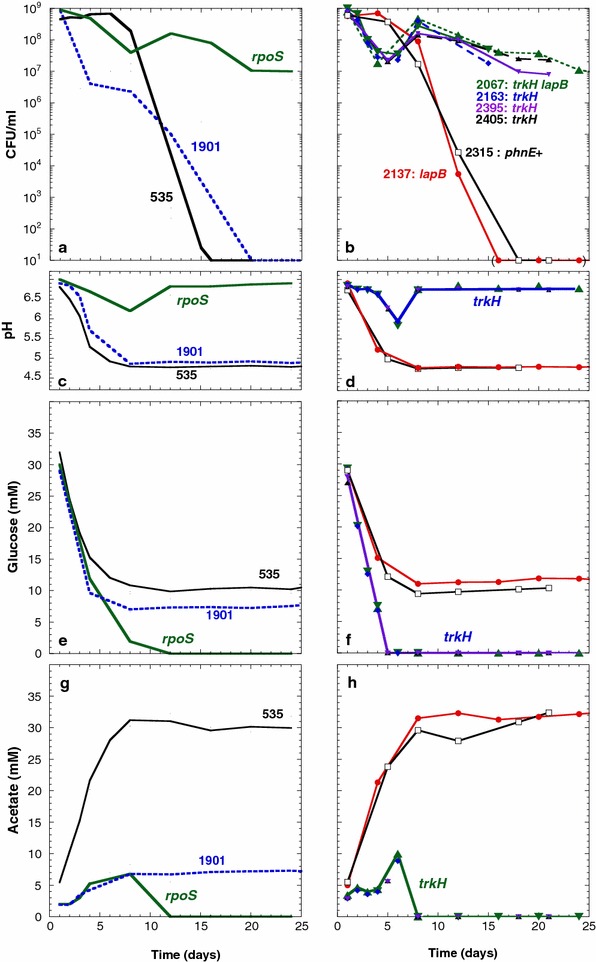
Similar effects of the *trkH80* and *rpoS*-null mutations on the viability and on the metabolic activity of strains incubated in monoculture. Strains were inoculated 1:500 (time zero) into Pi-limiting medium and further incubated. The numbers of CFU (**a**, **b**), the pH (**c**, **d**) and the concentrations of glucose (**e**, **f**) and of acetate (**g**, **h**) were determined between days 1 and 24 of incubation. The *curves* in panels **a**, **c**, **e**, **g** for the ancestral strain ENZ535, the evolved strain ENZ1901 and the *rpoS* strain were previously published [1]

Increased levels of K^+^ may inhibit the activity of proteins [35–37]. To determine whether the *trkH80* mutation could change the activity of enzymes involved in glucose metabolism, we determined the levels of glucose and of acetic acid in spent media during prolonged incubation of *trkH*^+^ and *trkH80* strains in Pi-limiting medium (Fig. 4). Surprisingly, the strains that harbored the *trkH80* mutation, independently of the nature of the *phnE* and *lapB* alleles (ENZ2163: *trkH80*; ENZ2395 and ENZ2405: *phnE*^+^*trkH80*; and ENZ2067: *phnE*^+^*trkH80 lapB43*), exhibited similar viability and metabolic pattern as single *rpoS* mutants during prolonged incubation in monocultures starved for Pi (Fig. 4a–h) [1]. First, *trkH80* mutant strains lost viability between days 1 and 6 of incubation (Fig. 4b), while glucose was completely consumed (Fig. 4f) and low levels of acetic acid were excreted (10 mM acetate at pH 6) (Fig. 4d, h). Second, by day 6 of incubation, cells grew on acetic acid (Ace^+^ phenotype), which was totally detoxified on day 8 of incubation (≤0.02 mM acetate at pH 6.8) (Fig. 4d, h). In contrast, the *phnE*^+^*lapB43* mutant strain (ENZ2137) behaved as the ancestral strain (ENZ2315: ENZ535 *phnE*^+^ and ENZ535) (Fig. 4a–h). Collectively, these results indicate that the *trkH80* mutation could change the metabolic flux in a similar manner to an *rpoS*-null mutation, but without affecting the activity of the RpoS factor.

### Effects of the *trkH80*, *lapB43* and *rpoS214* mutations in mixed cultures

**Fig. 5:**
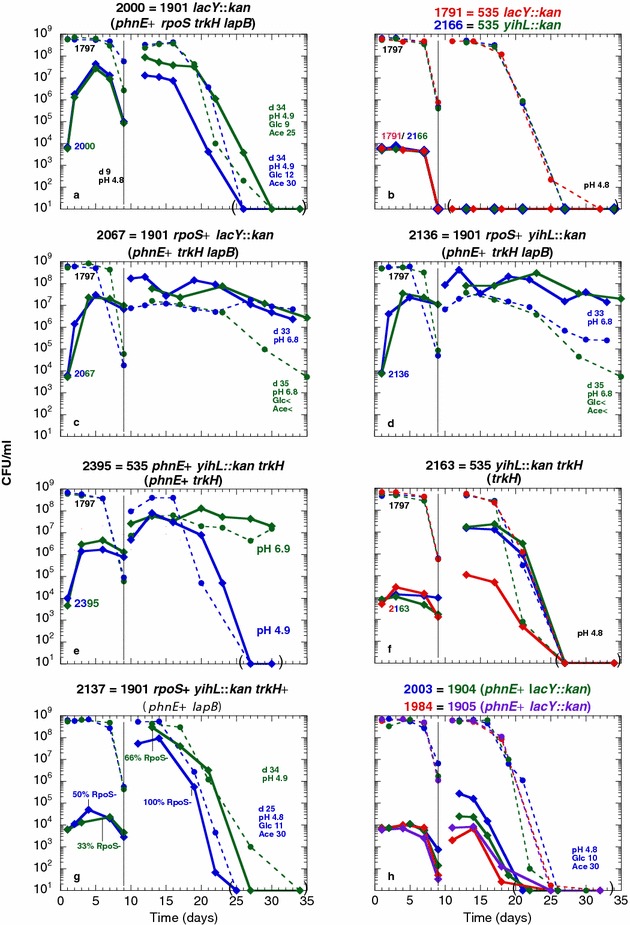
Effects of the *trkH80*, *rpoS214* and *lapB43* mutations on the viability of strains incubated in mixed culture. The strains tested as a minority (Kan^r^) were grown as monocultures in Pi-limiting medium for 1 day, diluted 10^5^-fold into 50 ml of 1-day-old cultures of the ancestral strain ENZ1797 (ENZ535 Tc^r^) in Pi-limiting medium, and incubated further for 8 days. On day 9 of incubation, 1 ml of mixed culture was added into 50 ml of fresh Pi-limiting medium and incubated further. At the end of the incubation period, the pH and the concentrations of glucose (Glc) and of acetate (Ace) were determined in spent media (concentrations are indicated in mM; the sign < indicates that the concentration was ≤0.02 mM). The viability of the ancestral strain (Tc^r^) is indicated with *dashed lines* of different *colours* for independent experiments. The viability of the test strains (Kan^r^) -ENZ2000 (**a**), ENZ1791 and ENZ2166 (**b**), ENZ2067 (**c**), ENZ2136 (**d**), ENZ2395 (**e**), ENZ2163 (**f**), ENZ2137 (**g**), and ENZ2003 and ENZ1984 (**h**)- is indicated with *solid lines*

To determine the effects of the mutations present in the evolved strain ENZ1901 during prolonged incubation in mixed culture, strains (Kan^r^) harboring different combinations of the wild type and evolved alleles were added as a minority in cultures of the ancestral strain (Tc^r^) starved for Pi, further incubated, and the co-cultures were re-diluted 1:50 into fresh Pi-limiting medium on day 9 of incubation and incubated up to day 35 (Fig. 5).

The efficiency of the GPS phenotype (determined on day 5 of incubation) decreased in the order: ≈5 × 10^7^ CFU/ml for *phnE*^+^*trkH80lapB43 rpoS214* mutants (ENZ2000) (Fig. 5a) [1]; ≈2 × 10^7^ CFU/ml for *phnE*^+^*trkH80lapB43* mutants (ENZ2067 and ENZ2136) (Fig. 5c, d); ≈2 × 10^6^ CFU/ml for *phnE*^+^*trkH80* (ENZ2395) (Fig. 5e); ≈7 × 10^4^ CFU/ml for *phnE*^+^*lapB43* mutants (ENZ2137) (Fig. 5g); ≈ 3 × 10^4^ CFU/ml for single *trkH80* mutants (ENZ2163) (Fig. 5f); and ≈10^4^ CFU/ml for single *phnE*^+^ (ENZ1984 and ENZ2003) (Fig. 5h) and parental strains (ENZ1791 and ENZ2166) (Fig. 5b) [1].

When the co-cultures were re-diluted into fresh medium, Kan^r^ strains were generally overtook by the ancestral strain (Tc^r^) and both strains lost viability owing to the high levels of acetic acid excreted (up to 30 mM acetic acid at pH 4.8, on day 34 of incubation). This notably occurred in the case of the evolved strain ENZ2000 (*phnE*^+^*trkH80lapB43 rpoS214*) (Fig. 5a). In sharp contrast, triple mutants *phnE*^+^*trkH80lapB43* (ENZ2067 and ENZ2136) overtook the ancestral strain, survived prolonged incubation, and afforded a robust cross-protection to the ancestral strain as a result of the detoxification of acetic acid in the incubation medium (≤0.02 mM glucose and acetate at pH 6.8, on day 35 of incubation) (Fig. 5c, d). The *phnE*^+^*trkH80* double mutants (ENZ2395) sometimes failed to overtake the ancestral strain, which then triggered the death of the whole population during prolonged incubation (Fig. 5e). The *phnE*^+^*lapB43* strain (ENZ2137) was overtook by the ancestral strain and died, whereas it rapidly evolved RpoS^−^ variants (Fig. 5g).

Therefore, the *lapB43* mutation, which had no effect on the metabolic pattern in monoculture (Fig. 4) and only a weak effect on the GPS phenotype, might improve the GPS phenotype and the long-term viability of *phnE*^+^*trkH80lapB43* triple mutants in mixed culture. In contrast, the addition of the *rpoS214* mutation into the *phnE*^+^*trkH80lapB43* strain slightly improved the GPS phenotype but dramatically decreased the long-term viability, which indicates that the presence of the *rpoS214* mutation in the evolved strain ENZ1901 (*phnE*^+^*trkH80lapB43 rpoS214*) was eventually deleterious.

### The *kdpD*-D460V mutation in ENZ1902 triggers a constitutive expression of the *kdpFABC* operon

**Fig. 6:**
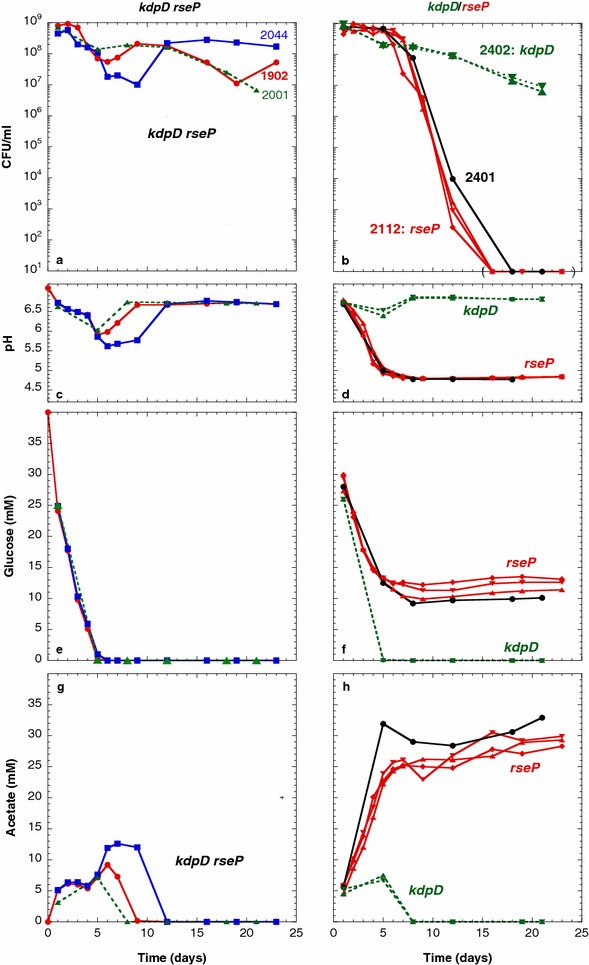
Effects of the *kdpD460* and *rseP98* mutations on the viability and on the metabolic activity of strains incubated in monoculture. Strains were inoculated 1:500 (time zero) into Pi-limiting medium, further incubated, and the numbers of CFU (**a**, **b**) in the cultures, the pH (**c**, **d**) and the concentrations of glucose (**e**, **f**) and of acetate (**g**, **h**) in the spent media were determined

**Fig. 7:**
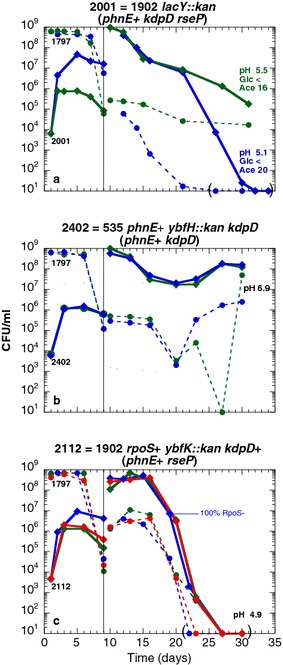
The *rseP98* mutation triggers a GPS phenotype but reduces the long-term viability of *kdpD460* mutants in mixed culture. The experiments were conducted as described in the legend to Fig. 5. The test strains (Kan^r^) are ENZ2001 (**a**), ENZ2402 (**b**), and ENZ2112 (**c**)

All phenotypic traits of the evolved strain ENZ1902 (*phnE*^+^*kdpD460rseP98*) matched with those of ENZ1901 *rpoS*^+^ derivatives (*phnE*^+^*trkH80lapB43*): Glg^−^, RpoS^+^ (Table 2), Ace^+^ in pure culture (Fig. 6a, c, e, g), and GPS^+^ in mixed culture (Fig. 7a). Therefore, we focused on the *kdpD460* mutation, which might affect K^+^ homeostasis like the *trkH80* mutation. KdpD belongs to the Kdp system composed of the high-affinity K^+^ transporter (KdpFABC) and of the two-component system KdpD/KdpE, in which KdpD is the sensor that signals low levels of K^+^ in the medium. Under K^+^-limiting conditions, KdpD phosphorylates KdpE, which induces the *kdpFABC* operon [32, 38].

**Fig. 8:**
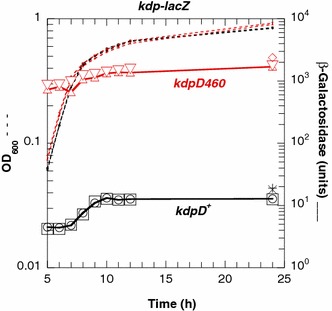
KdpD activity measured with the levels of expression of the P*kdp*::*lacZ* fusion in strains grown in Pi-limiting medium. The strains harbored the *kdpD* ^+^ [ENZ2337 (*circle*, *square*, *times symbol*); ENZ2363 (*plus symbol*)] and the *kdpD460* alleles [ENZ2360 (*triangle*, *inverted triangle*, *diamond*)]. The *symbols* represent independent experiments. The values were determined between 5 and 24 h of incubation (*circle*, *square*, *triangle*, *inverted triangle*) or after 24 h of incubation (*times symbol*, *plus symbol*, *diamond*)

To determine whether the *kdpD460* mutation could change the activity of the KdpD/KdpE two-component system, we used a strain carrying a P*kdpF*::*lacZ* fusion (ENZ2337) [14]. Transduction of the *kdpD460* allele into the reporter strain ENZ2337 increased by 100-fold the levels of β-galactosidase (Fig. 8); similar values are obtained when the reporter strain grows with limiting K^+^ concentrations [14]. These data indicate that the KdpD-D460V regulator was constitutively active, thereby mimicking the KdpD^Pi^ form normally present when cells are starved for K^+^.

**Fig. 9:**
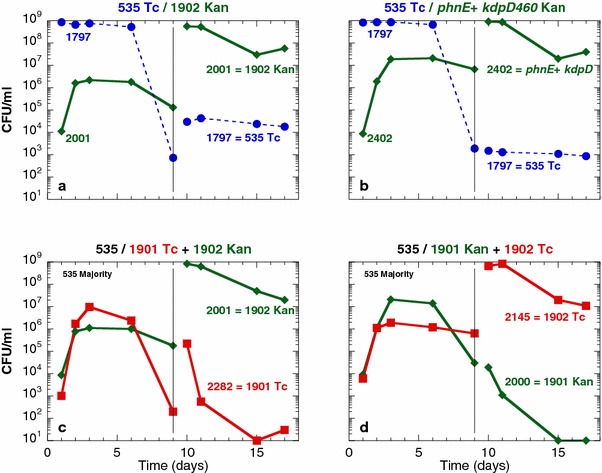
The *kdpD460* mutant strain prevents the growth of competing strains during prolonged incubation in mixed culture. The strains tested as a minority were grown as monocultures in Pi-limiting medium for 1 day, diluted 10^5^-fold into 50 ml of 1-day-old cultures of the ancestral strain in Pi-limiting medium, and incubated further for 8 days. On day 9 of incubation, 1 ml of mixed culture was added into 50 ml of fresh Pi-limiting medium and incubated further for up to 17 days. The ancestral strain was ENZ1797 (ENZ535 Tc^r^) when one evolved strain was added as a minority (**a**, **b**), and ENZ535 when two evolved strains were added as minorities (**c**, **d**)

The constitutive expression of the Kdp system might simply account for the new “growth inhibitory” (GI) phenotype exhibited by *kdpD460* mutant strains during prolonged incubation in mixed cultures. Upon dilution of 9-day old mixed cultures into fresh medium, the evolved strain ENZ2001 (*phnE*^+^*kdpD460rseP98* Kan^r^) (Figs. 7a, 9a) and the reconstructed strain ENZ2402 (*phnE*^+^*kdpD460* Kan^r^) (Figs. 7b, 9b) prevented growth of the ancestral strain ENZ1797 (ENZ535 Tc^r^). Moreover, the evolved strain ENZ1902 (*phnE*^+^*kdpD460rseP98*, Kan^r^ or Tc^r^) apparently starved to death the evolved strain ENZ1901 (*phnE*^+^*rpoS214 trkH80lapB43*, Tc^r^ or Kan^r^) when the two evolved strains were added as competing minorities in mixed cultures containing the ancestral strain ENZ535 in majority (Fig. 9c, d). In fact, the GI phenotype exhibited by *kdpD460* mutant strains might result from the scavenging of K^+^ through the constitutive activity of the high-affinity Kdp system, which might starve other strains in the population.

### The *rseP*-A98V mutation triggers a GPS phenotype

Compared to the evolved strain ENZ1902 (*phnE*^+^*kdpD460 rseP98*), the reconstructed strain ENZ2402 (*phnE*^+^*kdpD460*), which lacked the *rseP98* mutation, generally re-consumed acetic acid more efficiently in monoculture (Fig. 6g, h), and in mixed culture (Fig. 7a, b). These results suggest that in the evolved strain ENZ1902, the *kdpD460* mutation played a beneficial role, whereas the *rseP98* mutation could play a detrimental role in metabolism.

To further characterize the role of the *rseP98* mutation in metabolism, we exchanged the *kdpD460* mutant allele for the *kdpD*^+^ allele in ENZ1902, and tested the new strain, ENZ2112 (*phnE*^+^*rseP98*), in monoculture and in mixed culture. In monoculture, the strain ENZ2112 excreted somewhat less acetic acid and consumed less glucose than a *phnE*^+^ strain (Fig. 6f, h), which confirmed that the *rseP98* mutation might cause a slight defect in metabolism during prolonged incubation. Surprisingly, the strain ENZ2112 (*phnE*^+^*rseP98*) exhibited a strong GPS phenotype but did not survive prolonged incubation in mixed culture (Fig. 7c).

**Fig. 10:**
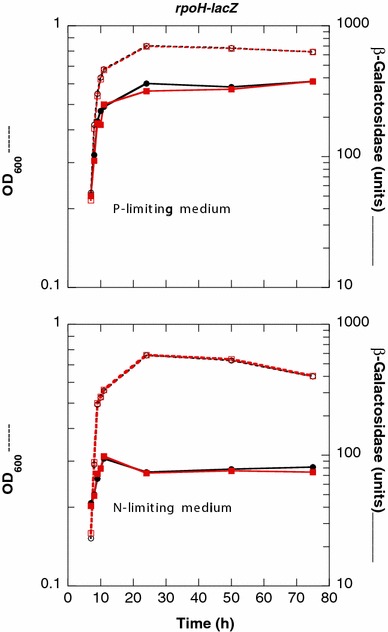
The RpoE activity measured with the levels of expression of the *rpoH*P3::*lacZ* fusion is higher in P- than in N-starved cells. The strain ENZ2489 (*phnE* ^+^ *rseP* ^+^) was inoculated 1:500 (time zero) into P- and N-limiting media and further incubated. The OD_600_ (*dashed lines*) and the β-galactosidase activity were determined (*solid lines*). The *symbols* represent two independent experiments

RseP is an inner-membrane protein. Its primary role is to contribute to the cleavage of the RseA anti-σ^E^ factor, which triggers the stress envelope σ^E^ (RpoE) regulon [39, 40]. To determine whether the *rseP98* mutation could affect the expression of the RpoE regulon in Pi-starved cells, we transduced the *rseP98* and *rseP*^+^ alleles into a reporter strain carrying the *rpoH*P3::*lacZ* fusion, which measured the activity of the σ^E^-RNA polymerase holoenzyme [7]. In an *rseP*^+^ derivative, the RpoE activity increased significantly at the approach of the stationary phase, especially under Pi starvation conditions (Fig. 10) [2, 7, 41]. A Δ*rseA* mutation increased by 3.8-fold the levels of expression of the *rpoH*P3::*lacZ* fusion in exponential (t = 7 h, OD_600_ = 0.25) and in stationary phases (t = 24 h) (Table 3), which indicates that the spontaneous induction of the RpoE regulon in Pi-starved cells was independent of the cleavage of RseA [7, 41]. A *rseP98* mutation had no effect on the levels of expression of the *rpoH*P3::*lacZ* fusion, neither in exponential growth phase nor in stationary phase (Table 3), which rules out a role of the RseP-A98V protein in the expression of the stress envelope σ^E^ (RpoE) regulon.

**Table 3:** The RpoE activity measured with the levels of expression of the *rpoH*P3::*lacZ* fusion is not affected by the *rseP98* mutation

ENZ strains	β-Galactosidase (units)	
	Exponential growth phase (7 h in P-limiting medium)	Stationary phase (24 h in P-limiting medium)
2333 (*rpoH*::*lacZ*)	37 ± 3 (n = 2)	277 ± 21 (n = 9)
2480: 2333 Δ*rseA*::*kan*	142 ± 1 (n = 2)	1043 ± 6 (n = 2)
2489: 2333 *yaeH*::*kan rseP* ^+^	39 ± 1 (n = 2)	282 ± 19 (n = 4)
2490: 2333 *yaeH*::*kan rseP98*	37 ± 1 (n = 2)	292 ± 3 (n = 4)

### Synergy of *arcA* and *rsxC* mutations on acetic acid detoxification in ENZ1903

**Fig. 11:**
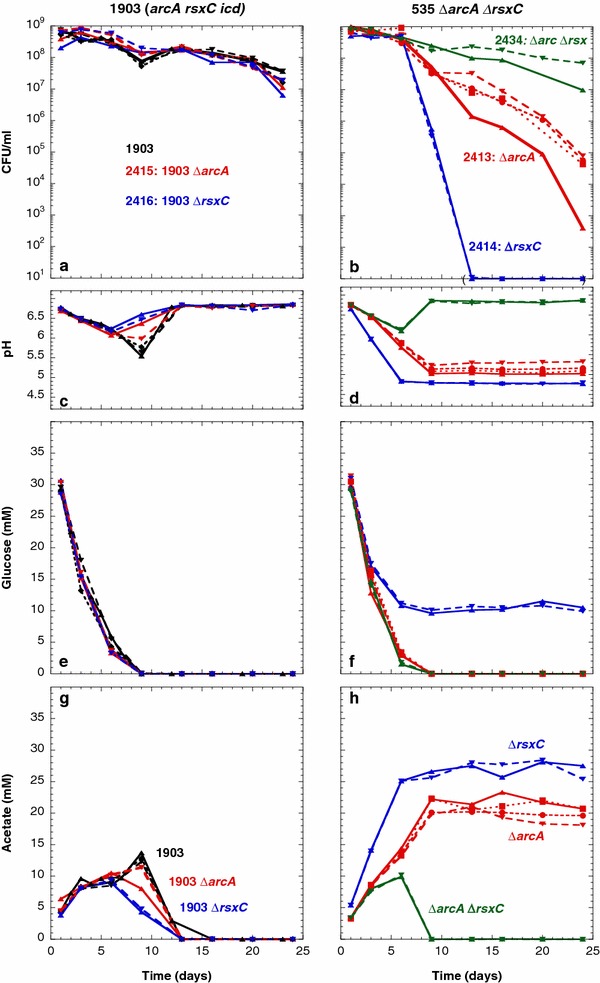
Synergistic effects of the *arcA* and *rsxC* mutations on the viability and metabolic activity of strains incubated in monoculture. The experiments were conducted as described in the legend to Fig. 4

**Fig. 12:**
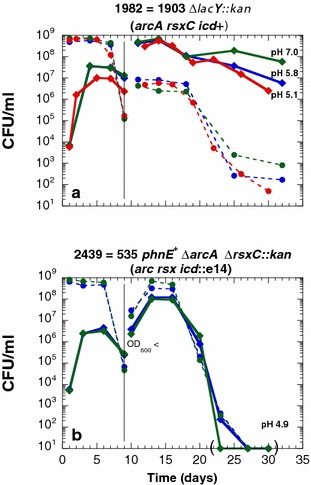
The *arcA* and *rsxC* mutations are not sufficient for a long-term viability in mixed culture. The experiments were conducted as described in the legend to Fig. 5. The test strains (Kan^r^) are ENZ1982 (**a**) and ENZ2439 (**b**)

Except for its Glg^+^ phenotype, the evolved strain ENZ1903 (*phnE*^**+**^*arcA*-F172Y *rsxC*-I525F e14^−^*/icd*^+^*crfC*-E693Stop) exhibited the characteristic phenotypes of *kdpD460* and *trkH80* mutant strains: Ace^+^ in monoculture (Fig. 11g), GPS^+^ (days 1–5) and long-term viability in mixed culture (Fig. 12a).

Mutations in *arcA,rsxC* and *icd* genes were not unexpected because they could affect the activity of the tricarboxylic acid cycle in different ways:

1. The dual regulator ArcA primarily inhibits the expression of genes implicated in the activity of the tricarboxylic acid cycle (e.g. *acnB* and *fumA*) and of the aerobic respiratory chain when the concentration of dissolved oxygen decreases. ArcA also increases the synthesis of CydAB, an enzyme of the aerobic respiratory chain that scavenges low levels of oxygen, which increases the energetic metabolism and extents the protection of cytoplasmic proteins (e.g. FNR) against oxygen at low pO_2_ [42, 43]. Inactivation of *arcA* increases the metabolic flux into the tricarboxylic acid cycle in exponentially growing cells [44].
2. RsxC belongs to the Rsx NADPH-dependent reducing system that inactivates SoxR and thus shunts off the SoxRS response to oxidative stress [45]. The SoxRS response comprises *acnA* and *fumC*, which encode proteins of the tricarboxylic acid cycle that are resistant to oxidative stress [46]. In exponentially growing cells, a Δ*rsxC* mutation increases the basal level of expression of the SoxRS regulon primarily because the [2Fe-2S] clusters in SoxR that are spontaneously oxidized cannot be re-reduced [45]. In addition, in *arcA* mutants, the oxygen-sensitive transcription factor FNR should be inactive, which might increase the cellular levels of SoxR and SoxS [42, 47].
3. During growth on acetate, the activity of Icd (isocitrate dehydrogenase) is decreased as a result of its phosphorylation, which helps to redirect part of the metabolic flux from the tricarboxylic acid cycle towards the glyoxylate shunt [11]. In *E. coli* K-12, the phage e14 is normally integrated in *icd* (Fig. 3). Although the sequences of *icd* in lysogenic (e14^+^; *icdA*) and in non-lysogenic strains (e14^−^; *icd*^+^) differ by 12 single-nucleotide polymorphisms, 10 synonymous and two non-synonymous, both forms of the Icd protein exhibit the same activity in vitro. Recent data suggest that excision of the prophage e14 and/or restoration of the *icd*^+^ gene could somehow decrease or increase the resistance of *E. coli* K-12 to different stresses [18, 19].

We found evidence that the *arcA*-F172Y allele encoded an inactive protein. First, we transduced a *cydA*::*lacZ* fusion, which is induced by ArcA [13], into the evolved strain ENZ1903 and into the ancestral strain ENZ535. After 24 h of incubation under aerobic, Pi starvation conditions, the levels of β-galactosidase were 3.4-fold lower in ENZ1903 (1787 ± 303 units, n = 2) than in ENZ535 derivatives (6062 ± 401 units, n = 2), which suggests that the evolved strain ENZ1903 might exhibit little, if any, ArcA activity [13]. Second, we exchanged the *arcA172* allele in ENZ1903 for a Δ*arcA*::*kan* deletion (ENZ2415), which did not change significantly the metabolic patterns of the strains in monocultures (Fig. 11a, c, e, g). Similarly, exchange of the *rsxC525* allele in ENZ1903 for a Δ*rsxC*::*kan* deletion (ENZ2416) had no significant effect on the metabolic patterns, which suggests that the *rsxC*-I525F mutation was null or neutral (Fig. 11a, c, e, g).

We transduced the Δ*arcA*::*kan* mutation (the kanamycin resistance marker could be eventually excised) and/or the Δ*rsxC*::*kan* mutation into the ancestral strain ENZ535, and determined the metabolic patterns of the different strains during incubation in monocultures (Fig. 11). Whereas the Δ*rsxC* mutation alone (ENZ2414) had no effect on the cellular viability and on the kinetics of production of acetic acid, the Δ*arcA* mutation alone (ENZ2413) had moderate effects: values were in-between the values obtained for the ancestral strain and the evolved strain ENZ1903 (Fig. 11b, d, f, h). Surprisingly, transduction of the Δ*rsxC*::*kan* mutation into the Δ*arcA* mutant strain (ENZ2434: ENZ535 Δ*arcA* Δ*rsxC*) dramatically improved the viability of the strain and its ability to consume the low levels of acetic acid that were previously excreted (Fig. 11b, d, f, h).

### A Δ*rsxC* mutation increases the SoxRS oxidative stress response in Δ*arcA* mutants

Could the synergy of the Δ*arcA* and Δ*rsxC* mutations be explained by an effect of the *rsxC* mutation on the SoxRS oxidative stress response? To answer this question, we transduced the Δ*arcA* and/or Δ*rsxC* mutations into the strain ENZ1843, which carries a *soxS*::*lacZ* fusion that is induced by SoxR in its active (oxidized) form [15], and we measured the β-galactosidase activity in exponentially growing cells (6 h of incubation; OD_600_ = 0.1) and in non-growing cells (24 h of incubation in P-limiting medium).

**Fig. 13:**
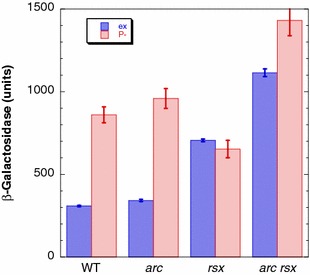
The combination of the *arcA* and *rsxC* mutations increases the levels of expression of the *soxS*::*lacZ* fusion. Strain ENZ1843 (*soxS*::*lacZ*) and derivatives ENZ2420 (Δ*arcA*), ENZ2421 (Δ*rsxC*) and ENZ2436 (Δ*arcA* Δ*rsxC*) were inoculated (0.1 ml) into 50 ml of Pi-limiting medium and β-galactosidase activities were determined in exponentially growing cells (6 h of incubation; OD_600_ = 0.1; n = 2) (ex) and in non-growing cells (24 h of incubation; n = 3) (P-). The values are the means ± standard deviations for n independent experiments

In exponentially growing cells, the *arcA* and *rsxC* mutations exhibited a synergistic effect on the levels of expression of *soxS*::*lacZ*, which increased barely in Δ*arcA* mutants, moderately in Δ*rsxC* mutants [45], and strongly in Δ*arcA* Δ*rsxC* double mutants (3.6-fold compared to wild type cells) (Fig. 13). Surprisingly, in Pi-starved cells, the levels of expression of *soxS*::*lacZ* changed in a complex manner (Fig. 13). In the parental strain ENZ1843 (WT), the levels of expression of *soxS*::*lacZ* were 2.8-fold higher in non-growing than in growing cells. We have previously shown that Pi-starved cells accumulate oxidative damage in macromolecules as a result of the endogenous production of H_2_O_2_ and HO^·^ radicals [4], which might oxidize guanine residues and consequently SoxR bound to the *soxS* promoter [48]. The levels of expression of *soxS*::*lacZ* further increased weakly in Δ*arcA* mutants, whereas they decreased in Δ*rsxC* mutants. The latter unexpected result might reflect the fact that in the absence of the RsxC activity, oxidized [Fe-S] clusters in SoxR may eventually lose Fe [45], which generates inactive apo-SoxR forms. Despite this caveat, the levels of expression of *soxS*::*lacZ* increased by 1.5-fold in Δ*arcA* Δ*rsxC* double mutants compared to Δ*arcA* single mutants (Fig. 13), which indicates that the Δ*rsxC* mutation could afford a protection to Δ*arcA* mutants starved for Pi through a higher level of expression of the SoxRS oxidative stress response.

### The *arcA* and *rsxC* mutations are not sufficient for a long-term viability in mixed culture

The constructed strain ENZ2439 (ENZ535 *phnE*^+^ Δ*arcA* Δ*rsxC*::*kan*), added in minority in a mixed culture, grew vigorously but died more rapidly than the evolved strain ENZ1982 (ENZ1903 Δ*lacY*::*kan*) between days 6 and 9 of incubation (Fig. 12a, b). Moreover, following the dilution of the mixed cultures into fresh medium on day 9 of incubation, the reconstructed strain ENZ2439 grew slowly and died off (Fig. 12b), whereas the evolved strain ENZ1982 overtook the population and survived prolonged incubation (Fig. 12a). A simple interpretation of these results is that the *icd*^+^ and/or *crfC693* alleles, present in the evolved strain (*phnE*^+^*arcA*172 *rsxC*525 e14^−^*/icd*^+^*crfC693*), but not in the reconstructed strain ENZ2439 (*phnE*^+^ Δ*arcA* Δ*rsxC icd*::e14), might help cells to tolerate a stress that built up in mixed culture.

## Discussion

It is generally thought that cells that evolve under nutrient limitation conditions primarily accumulate mutations that decrease the activity of the RpoS (σ^s^) factor [18, 20–22, 25, 26, 28, 29, 49]. We show here that in strains that evolved in serial batch cultures under aerobic, Pi starvation conditions, *rpoS*-null mutations, which were first beneficial, were eventually detrimental. In contrast, we identified novel combinations of mutations that were beneficial to the long-term viability of the cells.

### K^+^ homeostasis and metabolic flux

The *trkH80* and *kdpD460* mutations played a key role in the survival of the evolved strains ENZ1901 (*phnE*^+^*rpoS*-ΔG214 *trkH*-L80Q *lapB*-V43G) and ENZ1902 (*phnE*^+^*kdpD*-D460V *rseP*-A98V), respectively. The *trkH80* and *kdpD460* mutations triggered a constitutive expression of the low-affinity high-rate Trk system and of the high-affinity low-rate Kdp system, respectively.

The *trkH*-L80Q mutation was previously identified in strains that evolved in Pi-limited chemostat cultures and in serial batch cultures containing the antibiotic gentamicin sulfate [25, 50]. In the strain evolved in Pi-limited chemostat, the *trkH*-L80Q mutation was not further characterized because of the presence of numerous mutations [25]. In strains resistant to gentamicin, it was suggested that excess K^+^ could prevent the transport of the antibiotic inside the cells [50, 51]. The *kdpD*-D460V constitutive mutation was not previously described but the KdpD-D460V evolved protein is reminiscent of the KdpD-D474A engineered protein, which cannot sense K^+^ in the periplasm and thus constitutively activates the KdpE regulator of the *kdpFABC* operon [38].

How excess K^+^ could change the metabolism of glucose and of acetic acid in evolved strains? Potassium glutamate constitutes the most abundant ions inside the cell; K^+^ transport is linked to Pi transport and to internal pH (K^+^ transport inside the cell triggers a net H^+^ excretion) [32, 33, 36, 38, 52]. The activity of the K^+^ transporters can rapidly increase when the osmotic pressure of the medium increases and when the concentration of K^+^ in the medium decreases [32, 36], but excess K^+^ may be eventually toxic [32]. Interestingly, IlvBN, the first enzyme in the pathway of biosynthesis of branched-chain amino acids is especially sensitive to allosteric inhibition by K^+^ [37]. Because *ilvB* evolved through duplication/deletion events from *poxB* [53], it is tempting to speculate that both IlvB and PoxB might be inhibited by excess K^+^, which would direct the metabolic flux from pyruvate towards the tricarboxylic acid cycle rather than towards the synthesis of branched-chain amino acids and especially acetic acid in Pi-starved cells (Fig. 1).

### Changes in outer membrane protein activities

**Fig. 14:**
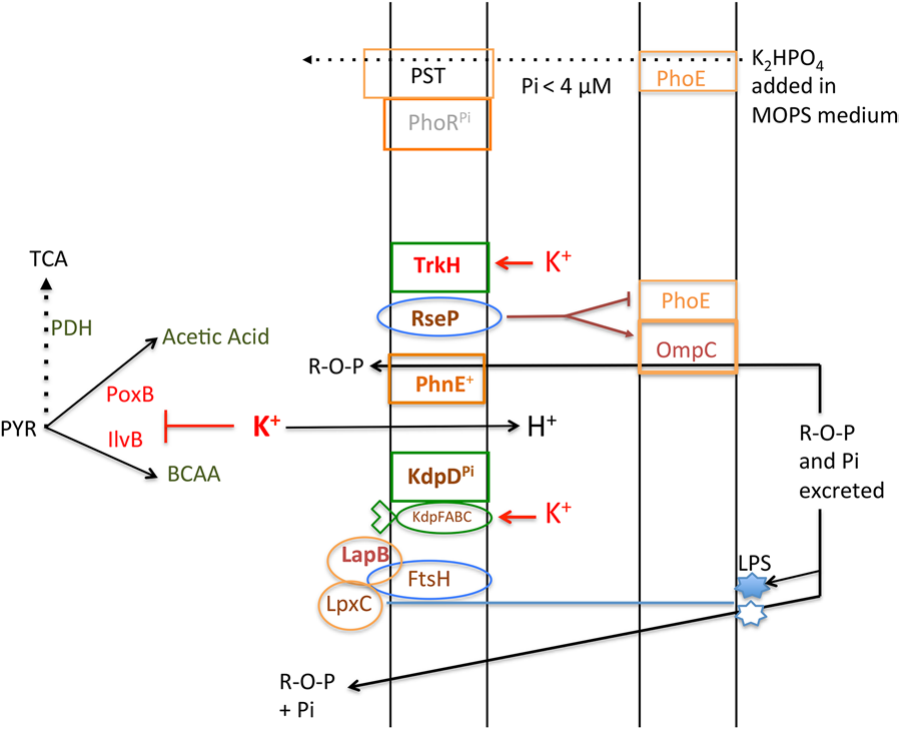
Schematic overview of possible changes in ENZ1901 and ENZ1902. *BCAA* branched-chain amino acids, *LPS* lipopolysaccharide, *PDH* pyruvate dehydrogenase, *PST* phosphate transport, *PYR* pyruvate, *R-O-P* organophosphate, *TCA* tricarboxylic acid

We found evidence that the *lapB43* mutation, present in the evolved strain ENZ1901, might help growth on glucose and organophosphates (GPS phenotype), and eventually on acetate and Pi (Ace^+^ phenotype). LapB is an inner membrane protein that negatively controls the biosynthesis of the lipopolysaccharide [30, 31]. Because the lipopolysaccharide is negatively charged as a result of the presence of two phosphates in lipid A [54], we suggest that the lipopolysaccharide might hamper the binding to the outer membrane of negatively charged products such as Pi and organophosphates. Therefore, we speculate that *lapB43* might be a gain-of-function mutation that would decrease the levels of the lipopolysaccharide, which might increase the binding and the diffusion of Pi and organophosphates required for the expression of the GPS phenotype (Fig. 14). The binding of P compounds to the outer membrane may be critical in *rpoS*^+^ cells in which the induction of the Pho regulon and the synthesis of P-compound scavengers are very transient [25]. In this light, the acquisition of the *rpoS214*-null mutation in the *phnE*^+^*lapB43* strain might favor the GPS phenotype in two different ways: by rerouting the metabolic flux towards the tricarboxylic acid cycle [1] and by extending the expression of the Pho regulon [25]. Unfortunately, the *rpoS214* mutation reduced the long-term viability of the evolved strain ENZ1901 in mixed culture; this may reflect a defect in acetic acid resistance through the so-called RpoS-dependent amino acid-independent “acid resistance system 1” [55].

The *rseP98* mutation, present in ENZ1902, triggered growth on glucose and organophosphates but hampered growth on acetic acid and Pi, which threatened the long-term viability of the cells in mixed culture. Although the primary role of RseP is to cleave the anti-sigma E factor, RseA [39], the *rseP98* mutation had no effect on the level of expression of the RpoE (σ^E^) regulon, which was normally induced in Pi-starved cells [5, 7]. Because inner membrane metalloproteins such as FtsH and RseP may exhibit dual functions in membrane protein biogenesis, as protease and as chaperone [56–58], it is tempting to speculate that the RseP-A98V protein might affect the folding of outer membrane proteins, which might increase the PhnE-dependent scavenging of organophosphates and decrease the PhoE-dependent scavenging of Pi (Fig. 14). Such trade-off would be reminiscent of the apparent changes in activities of the porins OmpC and PhoE, which occur in chemostat cultures limited in glucose [28].

### Resistance to oxidative and acetic acid stresses

The *arcA172* mutation might play a key role in the metabolic changes that occurred in the evolved strain ENZ1903. The global regulator ArcA progressively inhibits the transcription of genes of the tricarboxylic acid cycle when the concentration of dissolved oxygen decreases, which redirects the metabolic flux towards the production of weak acids such as acetic acid [44]: the inactivation of ArcA might thus maintain the activity of the tricarboxylic acid cycle in Pi-starved cells, which might decrease the production of acetic acid and allow its re-consumption (Fig. 1). Although different *arcA* mutations occurred in strains that evolved under nutrient stress conditions, the *arcA172* mutation, which affects the DNA-binding domain of ArcA, was not previously described [21, 22, 26].

A single Δ*arcA* mutation moderately decreased the production of acetic acid under aerobic, Pi starvation conditions, which suggests that the tricarboxylic acid cycle was poorly expressed. It is generally thought that the limited activity of the tricarboxylic acid cycle in *arcA* mutants might result from an insufficient activity of the aerobic respiratory chain, which would limit the re-oxidation of NADH; excess NADH may inhibit the activity of the citrate synthase, GltA, which controls the entry into the tricarboxylic acid cycle [11, 59, 60] (Fig. 1). As our results revealed novel synergistic effects between the Δ*arcA* and Δ*rsxC* mutations on the metabolism of acetate and on the expression of the SoxRS oxidative stress response, we would like to suggest that metabolic defects in Δ*arcA* mutants would result not from the inhibition of enzyme activities by excess NADH but rather from the degradation of enzymes of the tricarboxylic acid cycle (e.g. AcnB and FumA) by reactive oxygen species adventitiously produced by NADH dehydrogenases [4] (Fig. 1).

In mixed culture, the evolved strain ENZ1903 (*phnE*^+^*arcA172 rsxC525* e14^−^/*icd*^+^*crfC693*) survived better than the ENZ535 *phnE*^+^Δ*arcA* Δ*rsxC* reconstructed strain, which suggests that the e14^−^/*icd*^+^ and/or *crfC*693 genetic changes might protect the strain ENZ1903. Interestingly, strains evolving at pH 4.8 accumulate mutations in *icd*, including an *icd*-D398E mutation [61]. We realized that this mutation was identical to a single-nucleotide polymorphism induced by the excision of the prophage e14 from *icd*: the *icdA* (*icd*::e14) and *icd*^+^ (e14^−^) alleles differed by 12 single-nucleotide polymorphisms, 10 synonymous and 2 non-synonymous including the D398E change (Fig. 3). Although the enzymes encoded by the *icdA* and *icd*^+^ alleles exhibit the same activity in vitro, it is likely that active enzymes were produced at different levels in vivo as a result of changes in codon usage, which may affect the rate of synthesis and the folding of the proteins [62, 63]. We propose that the isocitrate dehydrogenase activity might increase as a result of the e14^−^*/icd*^+^ genetic changes, which might increase the levels of α-ketoglutarate, of glutamate (synthesized from α-ketoglutarate), and of NADH (produced by the NAD-dependent α-ketoglutarate dehydrogenase): glutamate may be used by the RpoS-dependent GadB decarboxylase [10], which may consume protons inside the cell, and the re-oxidation of NADH by the aerobic respiratory chain may trigger the excretion of protons (Fig. 1). Both processes might help the evolved strain ENZ1903 to resist internal acidification by acetic acid (pH 4.8) that was excreted into the medium by the ancestral strain present in majority in mixed culture between days 1 and 9 of incubation. In this light, it is tempting to speculate about how the truncation of CrfC (*crfC*-E693Stop), which normally tethers clamp-replication forks complexes [64], could favor the e14^−^/*icd*^+^ genetic changes: the CrfC693 mutant protein might destabilize the replication forks [64], generate excess single-stranded DNA, activate the RecA protein, and trigger the excision of the lambdoid prophage e14 (e14^−^) [65, 66].

### Parallel evolutions that change acetate producers in acetate consumers

Collectively, our results revealed that to survive during prolonged incubation under aerobic, Pi starvation conditions, evolved strains acquired several types of mutations that helped them to grow on glucose and organophosphates (GPS phenotype), to produce low levels of acetic acid, and to combat endogenous oxidative stress and exogenous acid stress. Then, evolved strains that overtook the population could grow on acetic acid and Pi previously excreted (Ace^+^ phenotype), thereby detoxifying the medium [5].

Based on the dynamics of evolving populations (generally, RpoS^−^ strains spread first, followed by Glg^−^ and eventually Glg^+^ strains) [1], we would like to suggest a possible scenario for the time course of spreading of evolving strains. It is likely that the *phnE*^+^ change was primarily selected because the 8-bp deletion in *phnE*, which results from a slippage between direct repeats during replication, occurs with a high frequency [1]. Then, acquisition of the *lapB43* and especially *rpoS214* mutations, which triggered growth and thus DNA replication under Pi starvation conditions, might increase the probability of acquiring the *trkH80* constitutive mutation, thereby generating the strain ENZ1901 that exhibited a high metabolic rate [1]. A similar evolutionary process might occur with the acquisition of the *phnE*^+^ and *rseP98* mutations, which triggered a GPS phenotype, and ultimately of the *kdpD460* constitutive mutation, thereby generating the strain ENZ1902. Although the evolved strains ENZ1901 (RpoS^−^) and ENZ1902 (Glg^−^) were eventually doomed to die, these strains could provide a transient protection to the population by consuming glucose while producing low levels of acetic acid, which could provide sufficient time for the progressive accumulation of the *arcA172* and *rsxC525* mutations, and then the *crfC93* and e14^−^*/icd*^+^ genetic changes, thereby generating the strain ENZ1903. Eventually, during prolonged incubation in detoxified spent medium at pH 7, *phnE*^+^ single mutant strains (ENZ1904 and ENZ1905) could sweep the population, thereby restoring the ancestral metabolism.

### Useful strains

Considering the characteristics of the evolved (ENZ1903) and reconstructed strains (*trkH80 lapB43* and *kdpD460*), we suggest that these strains could be useful in human health and in industrial production of bacterial products.

As previously suggested, *E. coli* strains that evolved under aerobic, Pi starvation conditions might help to colonize the human intestinal tract, in which both Pi and glutamate are reabsorbed [1]. In this light, it would be interesting to determine whether evolved strains could exhibit probiotic effects, alone or in consortium [67].

The consumption of energy and amino acids for growth and the generation of metabolic stresses (e.g. envelope stress) can limit the production and excretion of proteins and chemicals. To resolve these problems, it has been suggested to stop growth but not protein synthesis, and to use engineered strains (e.g. *ptaackApoxB and arcA iclR* mutants) that produce less acetic acid and are more tolerant to stress [68–71]. We would like to suggest that the evolved strain ENZ1903 (*phnE*^+^*arcA172 rsxC525 crfC693* e14^−^*/icd*^+^) incubated in batch culture under aerobic, Pi starvation conditions could afford a platform for producing toxic proteins and chemicals because of two major characteristics: (1) the sustained activity of the tricarboxylic acid cycle in stationary phase, which helps to produce energy and building blocks rather than acetic acid, and (2) the spontaneous induction of several stress responses, i.e. the SoxRS oxidative stress response and a novel e14^−^*/icd*^+^ acid resistance system, which are specifically induced in ENZ1903, in addition to the Cpx and RpoE envelope stress responses and the RpoS general stress response, which are normally induced in Pi-starved cells [2].

## Methods

### Strains

The *E. coli* K-12 laboratory strain MG1655 is very close to commensal strains [72]. Among several MG1655 isolates [17], we chose the strain CF1648, therein called ENZ535 (Table 1), because it exhibited a high growth rate and a high survival rate during prolonged incubation in rich medium, in glycerol-minimal medium under aerobic conditions, and in glucose-minimal medium under aerobic and anaerobic conditions [1, 10, 73]. Strains of the Keio collection [16] were used as donors to transduce single-gene in-frame deletions replaced with a kanamycin-resistance cassette (Kan^r^). Transductions were performed with phage P1*vir* and cells grown in LB_5_ medium containing 5 g/l NaCl [73, 74]. To construct Kan^s^ derivatives of deletion mutants, the *kan* cassette flanked by the FLP-recombination-target sites was removed by introducing the FLP recombinase-expressing plasmid pCP20 at 32 °C and purifying clones at 42 °C [16]. We co-transduced evolved mutations with Kan^r^ markers from the Keio collection: *trkH* with Δ*yihL*::*kan*, *kdpD* with Δ*ybfH*::*kan*, and *rseP* with Δ*yaeH*::*kan*. The Δ*lacIZ* mutants were constructed as previously described [10]. Spontaneous PhnE^+^ strains were selected on MOPS minimal medium plates containing 0.05 mM methyl phosphonate as the sole source of P [1].

Before we sequenced the genomes of the evolved strains, we exchanged *rpoS* alleles for *rpoS*^+^ in ENZ1901 and ENZ1902. Practically, we transduced the *cysC*::Tn*10* tetracycline-resistance marker (Tc^r^; 7 kb apart from *rpoS*, frequency of co-transduction of 35 %) from ENZ2005 (ENZ535 *cysC*::Tn*10* Δ*rpoS*::*kan*) into evolved strains, selected *cysC*::Tn*10* Δ*rpoS*::*kan* (Tc^r^ Kan^r^) strains, and transduced the *cysC*^+^*rpoS*^+^ region from the ancestral strain ENZ535 (Cys^+^ Kan^s^). The evolved strains ENZ1901 (RpoS^−^) gave rise to ENZ2043 (Glg^−^), and the evolved strains ENZ1902 (Glg^−^) to ENZ2044 (Glg^−^). In the latter case, the strains ENZ1902 and ENZ2044 were indeed identical (*rpoS*^+^) as revealed by sequencing of the genomes.

### Media and culture conditions

The MOPS minimal medium used for the pre-cultures contained notably 40 mM MOPS (pH 7.4), 86 mM NaCl, 9.5 mM NH_4_Cl, 20 mM glucose, and 5 mM K_2_HPO_4_. The P-limiting MOPS minimal medium contained 40 mM MOPS (pH 7.4), 86 mM NaCl, 9.8 mM KCl, 9.5 mM NH_4_Cl, 40 mM glucose, and 0.1 mM K_2_HPO_4_. The N-limiting MOPS minimal medium contained 40 mM MOPS (pH 7.4), 86 mM NaCl, 2 mM NH_4_Cl, 40 mM glucose, and 5 mM K_2_HPO_4_ [10].

To perform monocultures, strains grown for 24 h in MOPS medium were inoculated (0.1 ml) into 50 ml of P- or N-limiting medium in 500-ml Erlenmeyer flasks (time zero) and incubated with agitation at 150 rpm in a covered water bath rotary shaker at 37 °C. To perform mixed cultures, the strains tested as a minority were grown as monocultures in P-limiting medium for 1 day, diluted 10^3^-fold in MOPS_0_ buffer (MOPS medium without P, N and C source), added (0.5 ml) into 50 ml of 1-day-old cultures of the ancestral strain in P-limiting medium, and incubated for 8 days. On day 9 of incubation, 1 ml of mixed culture was added into 50 ml of fresh P-limiting medium and incubated further for up to 30 days. In order to distinguish the different strains in mixed cultures, we transduced the *lacY*::Tn*10* (Tc^r^) and Δ*lacY*::*kan* (Kan^r^) mutations [1].

### Determination of RpoS, Glg and PhnE phenotypes

The RpoS^−^ (Glg^−^ KatE^−^) and Glg^−^ (Glg^−^ KatE^+^) phenotypes were determined on isolated colonies grown on LB_10_ medium plates containing 10 g/l NaCl as previously described [1, 74]. The PhnE^+^ phenotype was determined on isolated colonies grown on glucose MOPS minimal medium plates containing 0.05 mM methyl phosphonate as the sole source of P [1].

### Measurement of cell viability

To assess cell viability, serial dilutions were prepared in M9 buffer and aliquots (10 μl) were spotted in triplicate onto LB_10_ medium plates, which were spread with 2000 units catalase and might contain 30 μg/ml kanamycin or 12 μg/ml tetracycline [1, 74]. In the figures, the values of 10 CFU/ml in parentheses indicate that no CFU were detected when 5 × 20-μl portions of the cultures were directly plated.

### Levels of glucose and of acetic acid

The pH of the culture supernatants were determined at 25 °C, adjusted to pH 7 and the concentrations of glucose and acetate were determined by enzymatic tests (R-Biopharm) [1].

### Measurement of β-galactosidase activity

The β-galactosidase activity was determined as previously described [73, 74]. Units of β-galactosidase were expressed per OD_600_ [74].

### DNA sequencing

Whole-genome sequencing was performed with genomic DNA isolated with the “Wizard Genomic DNA Purification Kit” (Promega). Genomic DNA library preparation (Insert size: ~330 bp; Tiles: 120), sequencing (“Kit v5”, Illumina) with the “Genome Analyzer GA-IIx Illumina” (74 cycles), and data analysis (CASAVA-1.8.2) were performed by Imagif (CNRS). Tablet (1.12.13.26) was used for the visualization of sequence assemblies [75].

Sequencing of the genes of interest in the evolved and constructed strains was performed from PCR fragments generated with the “PCR Master Mix” (Promega) from colonies. PCR products were purified using a “QIAquick” PCR purification kit (Qiagen) and sequencing was performed by Beckman Coulter Genomics.
